# Nonleukemic Ureteral Granulocytic Sarcoma Presenting with Unilateral Urinary Obstruction and Hematuria

**DOI:** 10.1155/2013/861232

**Published:** 2013-08-19

**Authors:** Ömer Acar, Tarık Esen, Tülay Tecimer, Mustafa Çetiner, Önder Peker, Ahmet Musaoğlu

**Affiliations:** ^1^Department of Urology, VKF American Hospital, Istanbul, Turkey; ^2^School of Medicine, Koc University, Istanbul, Turkey; ^3^Department of Pathology, VKF American Hospital, Istanbul, Turkey

## Abstract

Granulocytic sarcoma is an extramedullary tumor which is composed of myeloblasts and immature myeloid cells. It usually occurs in association with acute myeloid leukemia and most commonly involves skin, soft tissue, lymph nodes, bone, and periosteum. We report a case of isolated ureteral granulocytic sarcoma without hematologic manifestations. Our patient presented with bloody urine and left-sided lumbar pain. Preoperative clinical and radiologic features raised the suspicion of an upper urinary tract transitional cell carcinoma, and he was scheduled for nephroureterectomy. However, perioperative pathologic feedback and the unusual endoscopic appearance of the tumor altered our surgical strategy towards segmental ureterectomy and ureteroneocystostomy. Eventual pathologic diagnosis was granulocytic sarcoma of the ureter. Postoperative workup failed to demonstrate any sign of an accompanying hematologic disorder. He started receiving the chemotherapy protocol of acute myeloblastic leukemia. To our knowledge, this is the first documented case of nonleukemic ureteral granulocytic sarcoma which came to attention due to urologic complaints.

## 1. Introduction

Granulocytic sarcoma (GS) is an extramedullary tumor which is composed of myeloblasts and immature myeloid cells. It may either precede the onset of or occur concurrently with acute myeloid leukemia. Less commonly, it may represent the blastic transformations of other myeloproliferative diseases [1]. Skin, soft tissue, lymph nodes, bone, and periosteum are the most common sites of involvement [2, 3]. Genitourinary disease, especially in the absence of hematologic manifestations, is exceedingly rare. Herein, we report a case with nonleukemic granulocytic sarcoma of the ureter which came to attention due to unilateral hydroureteronephrosis and macroscopic hematuria.

## 2. Case Presentation

A 41-year-old male patient presented with bloody urine and left-sided lumbar pain. Apart from arterial hypertension, which is regulated by two antihypertensives, he denied having any systemic comorbidity. Otherwise, the past medical history was unremarkable. His physical examination findings were within normal limits. Laboratory studies revealed normal creatinine level (0.9 mg/dL) and a hemoglobin of 13.2 g/dL, hematocrit level of 37.4%, white blood cell count of 16.500 (77% neutrophils, 9.7% lymphocytes, 9.7% monocytes, 2.2% eosinophils, and 0.1% basophils), platelet count of 189.000, and INR (international normalized ratio) level of 0.92. No abnormal cells were detected in peripheral smear.

**Figure 1:**
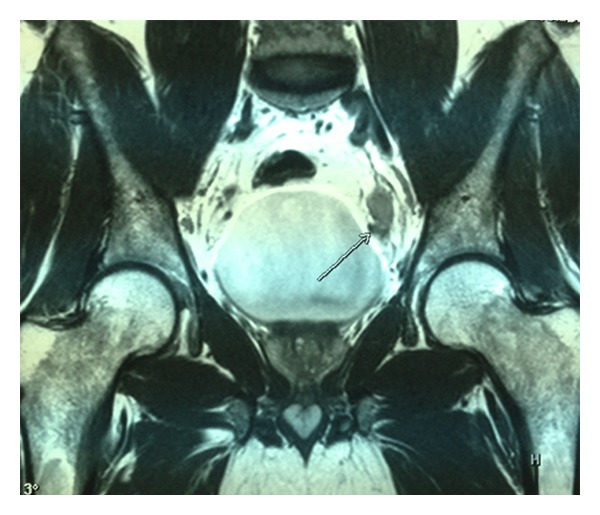
Coronal MR image demonstrating a solid mass located in the left distal ureter.

**Figure 2:**
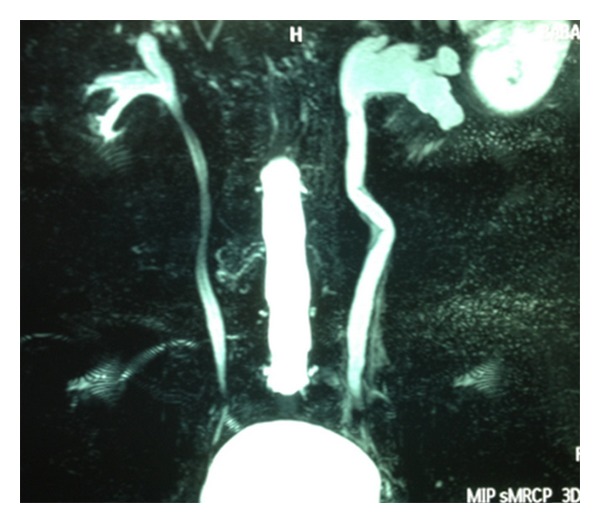
Coronal MR image demonstrating left-sided hydroureteronephrosis.

Magnetic resonance imaging (MRI) of the abdomen demonstrated a solid mass in the left distal ureter which extended between the iliac vessels and ureterovesical junction (Figure 1). Left pelvicalyceal system and proximal ureter were dilated due to the obstructive effect of this lesion (Figure 2). Additionally, there were lymph node enlargements in the external iliac, inguinal, and paraaortic regions. The largest lymph node was measuring 1 cm on the left paraaortic area. The presumptive initial diagnosis was transitional cell carcinoma (TCC) of the distal ureter based on these imaging findings. Therefore, we decided to perform diagnostic ureterorenoscopy and take biopsies from the tumoral lesion. According to the frozen-section findings, the definitive surgical intervention would be planned.

During ureterorenoscopy, we encountered a concentric mural thickening which started immediately after the intramural ureteral segment and projected upwards to involve the cranial 6 cm. The tumor did not exhibit a papillary configuration, and its rather infiltrative growth pattern had narrowed the ureteral lumen. We took cold-cup biopsies and sent them for pathologic evaluation. The pathologic examination was not informative due to the crush artefact. Neither the origin (TCC versus non-TCC) nor the nature (benign versus malignant) of the tumor could be identified. Considering the young age of the patient, the possibility of an eventual benign diagnosis, and feasibility of ureteral reimplantation after segmental surgery, we opted for distal ureterectomy and ureteral reimplantation. In addition, excised distal ureter would also be available for further frozen-section examination, upon which the natural course of the operation could be changed towards a nephroureterectomy.

**Figure 3:**
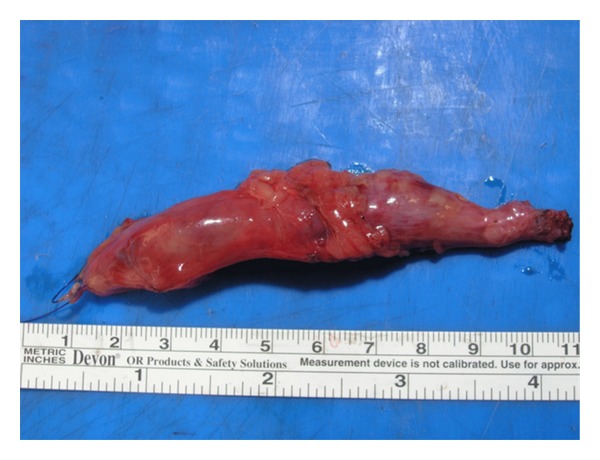
Macroscopic view of the excised ureteral segment.

Through a modified Gibson incision, we entered the retroperitoneal space and initially performed an extended lymphadenectomy. Afterwards, we dissected the tumor-bearing distal ureter down to the ureterovesical junction and excised a 10 cm long ureteral segment (Figure 3). Initial pathologic comment was in favor of a hematologic malignancy, though defining the exact subtype would need further immunohistochemical workup. Therefore, we decided to reimplant the remaining ureter using the psoas-hitch technique over a 4.8 Fr double-j catheter. After closing the mucosal defects, bladder wall was repaired in a triple-layered fashion. Bladder drainage was maintained with an 18 Fr urethral Foley catheter. After an uneventful postoperative period, the patient was discharged on day 4, and the urethral catheter was removed on day 7.

**Figure 4:**
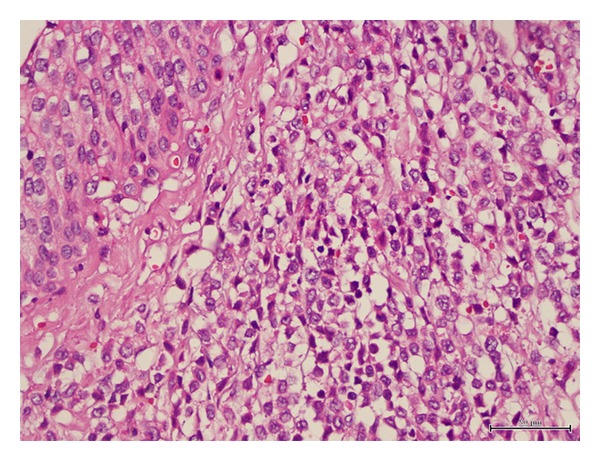
Neoplastic blasts located under the ureteral epithelium.

**Figure 5:**
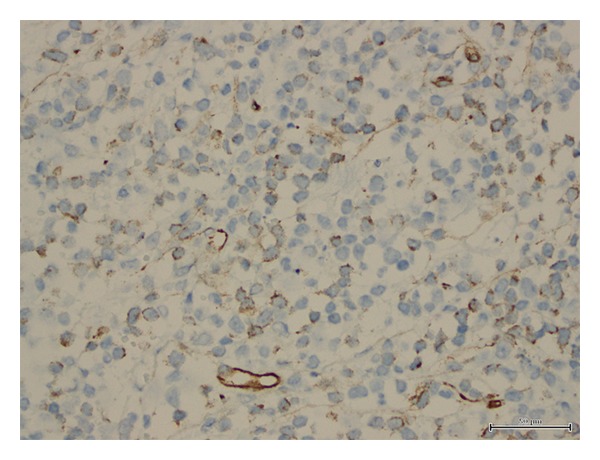
Neoplastic cells staining positive for CD34.

**Figure 6:**
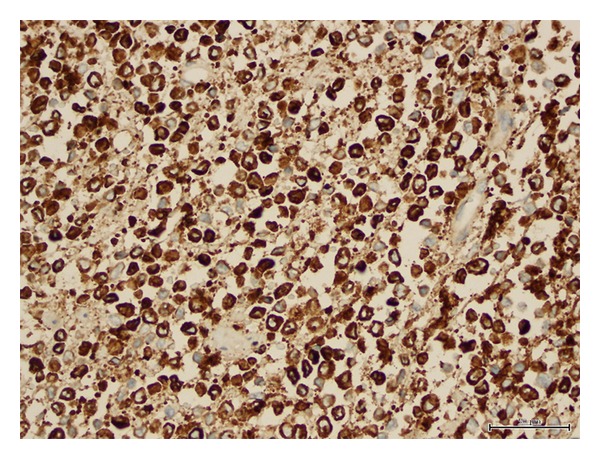
Neoplastic cells showing myeloperoxidase reactivity.

On pathologic examination, neoplastic cells showed diffuse infiltration of the whole ureteric wall (Figure 4). Immunohistochemically, they stained positive for CD34, myeloperoxidase, and CD43. CD68 and TdT reactions were partially positive. However, pan B-cell markers (CD20, Pax5) and pan T-cell markers (CD3) were negative. This immunohistochemical profile enabled us to categorize the neoplastic cells as myeloid blasts (Figures 5 and 6). Eventual pathologic diagnosis was myeloid sarcoma (granulocytic sarcoma) of the ureter, and the iliac lymph nodes were free of tumoral infiltration. Based on the histopathologic information, we evaluated our patient in terms of a possible concurrent hematologic malignancy (especially, acute myeloid leukemia). The distribution of the white blood cell population on the peripheral blood smear was as follows: 68% neutrophils, 19% lymphocytes, 9% monocytes, and 4% eosinophils, and the morphology of thrombocytes and erythrocytes was normal. Morphological, immunochemical, and flow cytometric evaluations of bone marrow were confirmed as normal. Finally, PET-CT scan ruled out the presence of a disseminated malignancy. After combining available data, our patient was diagnosed as having a nonleukemic form of granulocytic sarcoma which originated from the left distal ureter. He was scheduled for the classical consolidation regimen of acute myeloblastic leukemia, consisting of cytosine arabinoside 100 mg/m^2^/day for 7 days and idarubicin 12 mg/m^2^/day for 3 days.

## 3. Discussion

Granulocytic sarcoma is a localized extramedullary tumor which is composed of malignant cells of myeloid origin. Currently, GS is accepted as a part of the acute myeloid leukemia spectrum. Roughly it occurs in 2–8% of AML cases [3, 4]. It usually accompanies AML either at presentation or as a manifestation of recurrent disease. Less commonly, it may commence as a nonleukemic disease and end up with AML. When GS is an antecedent event, after a median period of 5 months the disease process will eventually evolve into AML [5]. Blastic transformations of other myeloproliferative or myelodysplastic diseases may also be seen in the form of granulocytic sarcoma [3].

Depending on the level of cellular maturity, blastic, immature, and differentiated subtypes have been defined for proper histopathologic classification [6]. An extramedullary tumor which is harboring poorly differentiated blasts and immature cells should raise the suspicion of GS. However, accurate pathologic diagnosis of GS is quite challenging, and in the past, many cases have been misdiagnosed as large cell lymphoma, Burkitt's lymphoma, lymphoblastic lymphoma, or undifferentiated round cell tumor [3, 4]. Imprint smears, flow cytometry and immunohistochemical studies provide useful discriminative information.

Granulocytic sarcoma is most commonly encountered in subperiosteal bone structures, lymph nodes, and skin [3]. Genitourinary tract is seldomly affected by this disease. The available literature consists of case reports, and the true incidence of genitourinary granulocytic sarcoma is unknown. Kidney, bladder, prostate, testes, epididymis, and uterine cervix are among the documented sites of genitourinary involvement [7–12]. Patients with lower urinary tract granulocytic sarcoma have usually presented with signs and symptoms related to bilateral urinary tract obstruction [8, 12] or urinary retention [9] and were often associated with AML either at diagnosis or at relapse [9, 11]. Unilateral ureteral involvement with no evidence of an accompanying hematologic abnormality has not been reported so far. Additionally, it is not uncommon for granulocytic sarcoma patients to be misdiagnosed as having another hematologic [7] or urothelial malignancy [8] and treated accordingly. In our case, although the radiologic picture was suspicious for an urothelial malignancy, frozen-section results of both the ureteroscopic biopsy and the ureterectomy materials kept us away from performing a nephroureterectomy. By doing so, we avoided rendering our patient with a single kidney, which is a significant concern given the fact that chemotherapy is the mainstay of treating his disease.

## 4. Conclusion

To our knowledge, this case represents the first report of nonleukemic, ureteral granulocytic sarcoma which presented with unilateral hydroureteronephrosis and hematuria. Histopathologic workup is crucial to the diagnosis and management strategy of these patients. Our surgical preference, which was based on the frozen-section data, will probably have a positive influence regarding chemotherapy compliance and renal functional prognosis.
